# Supplementary material for: Epidemiology of ankle sprains and anterior cruciate ligament injuries in youth basketball athletes in Niigata, Japan: A regional survey on injury management and healthcare professional involvement
Source: PLoS One. 2026 Mar 12;21(3):e0344196. doi: 10.1371/journal.pone.0344196 (PMC12981472; doi:10.1371/journal.pone.0344196)
Supplement: S2 Checklist — (DOCX) [file pone.0344196.s002.docx]

STROBE Statement—Checklist for Cross-sectional Studies

| Item No. | Recommendation | Brief Description | Location in Manuscript |
| --- | --- | --- | --- |
| 1 | Title and abstract | Indicate the study’s design in the title or abstract. | Title: '...A regional survey...', Abstract: 'A cross-sectional survey was conducted' |
| 2 | Background/rationale | Explain the scientific background and rationale for the investigation. | Introduction section, paragraph 1-3 |
| 3 | Objectives | State specific objectives, including any prespecified hypotheses. | End of Introduction |
| 4 | Study design | Present key elements of study design early in the paper. | Methods section: 'cross-sectional study' |
| 5 | Setting | Describe the setting, locations, and relevant dates. | Participants and Methods |
| 6 | Participants | Give eligibility criteria, and methods of selection. | Participants and Methods |
| 7 | Variables | Clearly define all outcomes, exposures, and confounders. | Assessment items |
| 8 | Data sources/measurement | For each variable, give sources and details of methods of assessment. | Assessment items |
| 12 | Statistical methods | Describe all statistical methods. | Statistical analysis: Chi-square test, R software |
| 13 | Participants | Report numbers of individuals at each stage of study. | Results: 'Valid responses were obtained from 2,747 participants' |
| 14 | Descriptive data | Give characteristics of study participants. | Table 1 |
| 15 | Outcome data | Report numbers of outcome events or summary measures. | Table 2 |
| 19 | Limitations | Discuss limitations of the study. | Limitations section |
| 21 | Generalisability | Discuss the generalisability of the study results. | Limitations and Discussion section |
| 22 | Funding | Give the source of funding. | Not explicitly stated; may need to be added if applicable. |
