# Supplementary material for: Epidemiology of ankle sprains and anterior cruciate ligament injuries in youth basketball athletes in Niigata, Japan: A regional survey on injury management and healthcare professional involvement
Source: PLoS One. 2026 Mar 12;21(3):e0344196. doi: 10.1371/journal.pone.0344196 (PMC12981472; doi:10.1371/journal.pone.0344196)
Supplement: Attachment — Submitted filename: Response to Reviewers.docx [file pone.0344196.s005.docx]

Response to Reviewers for manuscript PONE-D-25-32088

“Epidemiology of ankle sprains and anterior cruciate ligament injuries in youth basketball athletes in Niigata, Japan: A regional survey on injury management and healthcare professional involvement”

We would like to sincerely thank the Editor and Reviewers for their careful evaluation of our manuscript and for the constructive and insightful comments provided. We have carefully revised the manuscript in accordance with all suggestions and believe that these revisions have significantly improved the clarity, readability, and scientific rigor of the study.

In the revised version, we have addressed each comment in detail. Revision points include clarification of terminology for an international readership, improvements to language and tense consistency throughout the manuscript, refinement of interpretation to ensure appropriate caution, and enhancements to table formatting and overall presentation.

Below, we provide a detailed, point-by-point response to each reviewer comment. All corresponding changes in the manuscript have been highlighted.

- Judo Therapist Definition: Since PLOS ONE has a global audience, confirm what the role of a "Judo therapist" clearly defined as a nationally certified professional in Japan at its first mention in the results. I am not familiar with this term or profession.

**Response:** Thank you for pointing this out. We agree that the term may be unfamiliar to an international readership. We have therefore defined “judo therapist (judo seifukushi)” at its first mention in the Results and also added a brief definition in the Methods. Specifically, we now clarify that judo therapists are nationally licensed practitioners in Japan (licensed by the Ministry of Health, Labor and Welfare) who provide non-surgical management of acute musculoskeletal injuries (e.g., sprains, contusions, muscle strains) and that a physician’s consent is required for non-emergency treatment of fractures and dislocations (except for emergency first aid).

- Table Formatting: Verify that the formerly large "Table 2" has been successfully split into smaller, more readable tables (e.g., separate tables for incidence, medical consultation, and CAI/ACL prevalence) as requested by reviewers

**Response:** Thank you for this helpful suggestion. We carefully considered splitting the original Table 2 into multiple smaller tables. However, because the variables (injury incidence, healthcare professional consultation, and CAI/ACL prevalence) represent interconnected aspects of the same epidemiological framework, we retained a single integrated table to preserve interpretability and facilitate direct comparison across variables.
To improve readability as suggested, we have:

- clarified section headings within the table,
- improved column alignment and spacing, and
- added clearer subheadings to distinguish each domain.

We believe these revisions enhance readability while maintaining the logical integrity of the data presentation.

- Language and Flow: Please get an English editor as the tenses appear to be incorrect.

**Response:** Thank you for this important comment. We have revised the manuscript for English language, clarity, and overall flow, with particular attention to tense consistency throughout the Abstract, Methods, Results, and Discussion. We also performed a full proofread of the manuscript and corrected grammatical issues and awkward phrasing. These edits were made across the manuscript to improve readability for an international audience.

We also standardized tense usage so that the Methods and Results are consistently written in the past tense, while established knowledge in the Discussion is described in the present tense.

- CAI criteria in U-12 players: Although justified, applying adult criteria to children may still be questioned. The manuscript now notes this, but interpretation should remain cautious.

**Response:** Thank you for this important comment. We agree that applying CAI criteria originally developed for adult populations to younger athletes requires careful interpretation. We have therefore added clarifying statements in the Discussion and Limitations sections emphasizing that CAI prevalence estimates in the U-12 group should be interpreted with caution, considering potential developmental differences that may affect symptom reporting and classification.
